# Tubercular Otitis Externa in an Elderly Male- A Case Report

**Published:** 2019-03

**Authors:** Santosh-Kumar Swain, Mahesh-Chandra Sahu

**Affiliations:** 1 *Department of Otorhinolaryngology, IMS and SUM Hospital, Siksha“O”Anusandhan (Deemed to be University), K8, Kalinganagar,Bhubaneswar-751003, Odisha, India.*; 2 *Medical Research Laboratory, IMS and SUM Hospital, Siksha “O” Anusandhan (Deemed to be University), K8, Kalinganagar, Bhubaneswar-751003, Odisha, India.*

**Keywords:** Antitubercular therapy, External auditory canal, Otitis externa, Tuberculosis

## Abstract

**Introduction:**

Tuberculosis is one of the most common diseases in developing countries, resulting in significant morbidity and mortality. Tuberculosis has varied clinical presentations, varying from common primary pulmonary tuberculosis to the extremely rare tubercular otitis externa, as in this case. Tubercular otitis externa has an extremely low clinical incidence.

**Case Report:**

We report the case of an immunocompetent elderly male with chronic otorrhea, otalgia, and pale granulation tissue at the ear canal with a positive biopsy report for tuberculosis, confirming the diagnosis. Subsequently, sputum culture positive for Mycobacterium tuberculosis indicated disseminated tuberculosis. The patient’s symptoms resolved after antitubercular therapy (ATT).

**Conclusion:**

Tuberculosis at a rare location such as the external auditory canal is possible in regions like India where tuberculosis has the highest burden in the world. In the case of chronic ear discharge resistant to routine antibiotic treatment, the clinician should not rule out suspicion of tuberculosis.

## Introduction

Tuberculosis is a chronic infectious disease of humans caused by Mycobacterium tuberculosis, and has been known since the time of Hippocrates. Tuberculosis is an infection caused by intracellular acid-fast bacilli, demonstrated by different acid-fast stains such as kinyoun and the commonly used Ziehl-Neelsen staining (1). Tuberculosis affecting areas of the body other than the lungs is called extrapulmonary tuberculosis. Extrapulmonary tuberculosis is not uncommon and can occur in the head and neck region.

Tuberculosis has worldwide presence, and no organ of the human body is immune to it. The most common site is the lungs, while approximately 10% of tuberculosis cases have head and neck manifestations (2). Other than the neck nodes and larynx, tuberculosis of the head and neck region make up 2–6% of extrapulmonary and 0.1–1% of all forms of tuberculosis (3). Extrapulmonary tuberculosis has a prolonged clinical course and is often difficult to diagnose. Isolated tubercular otitis externa affects the external auditory canal without involving the lungs or any other organs of the body. Isolated tubercular otitis externa has an extremely low clinical incidence. Most clinicianS do not consider tuberculosis in their differential diagnosis when a patient presents with otorhinological symptoms, so often miss diagnosis leading to inappropriate treatment (4). A high index of suspicion is needed when a patient presents with otorhinolaryngeal tuberculosis, as it often runs an indolent course and its presentations can vary according to the anatomical site and adjacent areas. Here we present the case of a 72-year-old male with chronic left ear discharge subsequently diagnosed as otitis externa caused by Mycobacterium tuberculosis.

## Case Report

A 72-year-old male attended the otorhinolaryngology outpatient department with complaints of right ear discharge over 2 years. He had no other constitutional symptoms. The patient had undergone left side myringoplasty 5 years previously for chronic suppurative otitis media. His chronic ear discharge was not resolved after ear surgery. The ear discharge was sent for bacterial culture which showed no growth. The patient was taking topical ciprofloxacin ear drops, without any resolution of the symptoms.

**Fig 1:**
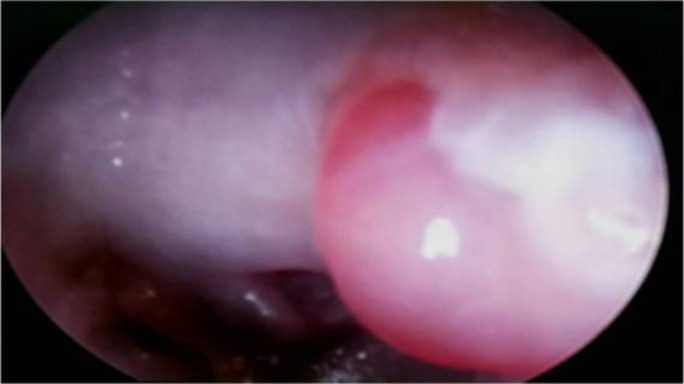
Tubercular lesions at the posterior superior part of the external auditory canal (arrow mark)

On examination of the ear, it revealed mild myringitis, thickening of the posterior ear canal, and active purulent discharge. There was some pinkish-to-pale colored granulation tissue at the posterosuperior quadrant of the tympanic membrane (Fig.1). 

**Fig 2:**
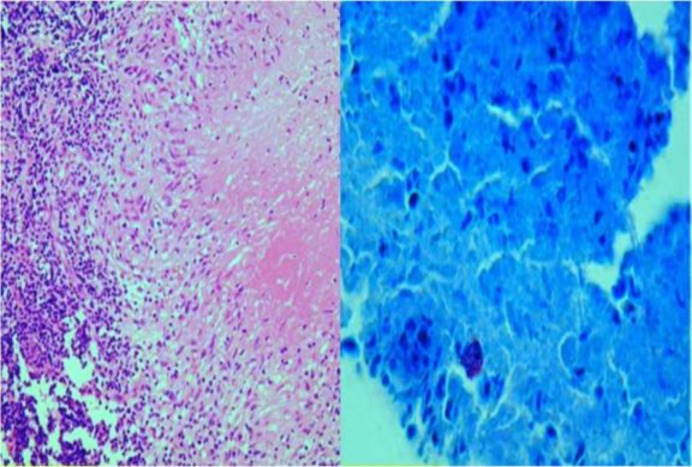
(a) Histopathological examination showing granuloma formation with caseous necrosis; (b) Ziehl-Nielsen staining from biopsy specimens showing acid-fast bacilli

The tympanic membrane was intact. The patient was concerned about malignancy and we took tissue from the granulations at the ear canal and sent it for histopathological examination. Histopathological examination showed squamous epithelium with granulo- matous inflammation. The acid-fast bacilli stain was positive for acid-fast bacilli (Fig.2). 

There were no fungal hyphae on Grocott’s methenamine silver stain and no evidence of any malignancy. Real-time polymerase chain reaction (PCR) of the formalin-fixed paraffin-embedded tissue was positive for Myco-bacterium tuberculosis, which was surprising.

**Fig 3:**
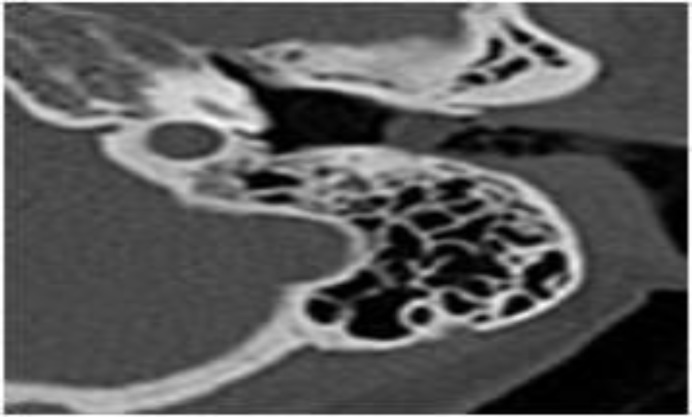
CT scan showing narrowing of the left external auditory canal

The patient was then properly evaluated by chest physicians, and denied any fever, cough, weight loss, or hemoptysis. He had no past history of exposure to tuberculosis. The patient’s routine laboratory findings including complete blood count, liver function tests, erythrocyte sedimentation rate (ESR), and serum creatinine were normal. However, QuantiFERON-TB Gold was positive. A computed tomography (CT) scan of the both sides of the temporal bone was performed, which showed narrowing of the left external auditory canal (Fig.3). 

This lesion was treated for extrapulmonary tuberculosis, including rifampicin (600 mg daily), isoniazid (300 mg daily), pyrazinamide (2,000 mg) daily, and ethambutol (1600 mg) daily for 2 months. The isoniazid and rifampicin were continued for the next 8 months. The patient’s otorrhea was resolved within 1 month after starting the antitubercular therapy (ATT). Examination of the external auditory canal and tympanic membrane were normal after 6 months of treatment.

## Discussion

Tuberculosis is a chronic granulomatous disease caused by Mycobacterium tuberculosis. It is one of the most prevalent diseases seen developing countries such as India. The incidence of tuberculosis is increasing in developing and in some developed countries of the world. The World Health Organization Statistical Information System (WHOSIS) has documented that developing countries like India have the highest incidence of tuberculosis in the world (5). Approximately 25% of these patients present extrapulmonary tuberculosis, of which 10–35% is found in the head and neck area (6). Otitis externa due to Mycobacterium tuberculosis is extremely rare, and the diagnosis of chronic otitis externa by tuberculosis in an immunocompetent adult without pulmonary involvement is rarely anticipated in clinical practice. There are, however, some cases of tubercular otitis media documented in the medical literature (7). Most of the cases of tubercular otitis media present with chronic ear discharge, otalgia, hearing loss, and facial nerve palsy (8). Our case was operated on in the past for bacterial otitis media without any evidence of Mycobacterium tuberculosis. The otitis externa was initially treated for bacterial infection, and was not healed. The chest X-ray of the patient was normal, although the sputum was positive for tuberculosis. The most likely pathogenesis for otitis externa was tuberculosis of the airway or nasopharynx with extension into middle ear via the Eustachian tube. The progression of the disease may spread to the external auditory canal. Definitive diagnosis is achieved by biopsy from the ear canal, which should be conducted before carrying out imaging so as not to disturb the continuity of the lesion.

Granulomatous inflammation with caseous necrosis and epithelioid giant cells are pathological findings in tuberculosis. An isolated tubercular lesion at the external auditory canal is a rare clinical incidence, even in the endemic zone of the tuberculosis (9). Histopathological examination shows granulomatous inflammation with epithelioid giant cells and caseous necrosis. Ziehl-Neelsen staining often directly detects acid-fast bacilli. The PCR test is also used for diagnosis of nasopharyngeal tuberculosis. In case of a strong clinical suspicion of tuberculosis and negative cultures, samples should be sent for PCR testing (2). CT and magnetic resonance imaging (MRI) are also valuable for imaging in head and neck tuberculosis. CT and MRI help to demonstrate the sites, extension, and pattern of the disease (10).

A diagnosis of isolated tuberculosis lesions at the external auditory canal was rare and surprising. In our case, the patient had no history of fever, cough, weight loss, or hemoptysis. His routine blood investigations including complete blood count, liver function test, and serum creatinine were within normal limits. Human immunodeficiency virus (HIV) serology testing of this patient was negative. The QuantiFERON-TB Gold in tube testing for latent tuberculosis was positive.

Management is ideally medical treatment with ATT including regimens of isoniazid (300 mg), rifampicin (450 mg), ethambutol (800 mg) and pyrazinamide (750 mg). Our case responded well to ATT. Early diagnosis and treatment in this case is paramount to help to prevent morbidity and complications. Therefore, if treated properly and early, this condition carries excellent prognosis and allows complete resolution of the tubercular otitis externa.

## Conclusion

Tuberculous otitis externa is a rare clinical entity requiring a multidisciplinary approach. Patients need standard ATT, even if they are immunocompetent. This case highlights the rare clinical presentation of tuberculosis and brings attention to a rare location of tuberculosis, which is possible in regions like India with the highest burden of tuberculosis in the world. In case of chronic ear discharge resistant to routine antibiotic treatment, clinicians should not rule out suspicion for tuberculosis.
